# Grafting of cationic molecules to hyaluronic acid improves adsorption and cartilage lubrication†

**DOI:** 10.1039/d4bm00532e

**Published:** 2024-08-08

**Authors:** Gavin Gonzales, Jiaul Hoque, Colin Kaeo, Stefan Zauscher, Shyni Varghese

**Affiliations:** a Department of Biomedical Engineering, Duke University Durham NC 27710 USA shyni.varghese@duke.edu +1-919-660-5273; b Department of Orthopaedic Surgery, Duke University School of Medicine Durham NC 27710 USA; c Department of Mechanical Engineering and Materials Science, Duke University Durham NC 27710 USA

## Abstract

Synovial fluid lubricates articular joints by forming a hydrated layer between the cartilage surfaces. In degenerative joint diseases like osteoarthritis (OA), the synovial fluid is compromised, which leads to less effective innate lubrication and exacerbated cartilage degeneration. Studies over the years have led to the development of partially or fully synthetic biolubricants to reduce the coefficient of friction with cartilage in knee joints. Cartilage-adhering, hydrated lubricants are particularly important to provide cartilage lubrication and chondroprotection under high normal load and slow speed. Here, we report the development of a hyaluronic acid (HA)-based lubricant functionalized with cationic branched poly-l-lysine (BPL) molecules that bind to cartilage *via* electrostatic interactions. We surmised that the electrostatic interactions between the BPL-modified HA molecules (HA-BPL) and the cartilage facilitate localization of the HA molecules to the cartilage surface. The number of BPL molecules on the HA backbone was varied to determine the optimal grafting density for cartilage binding and HA localization. Collectively, our results show that our HA-BPL molecules adhered readily to cartilage and were effective as a lubricant in cartilage-on-cartilage shear measurements where the modified HA molecules significantly reduce the coefficient of friction compared to phosphate-buffered saline or HA alone. This proof-of-concept study shows how the incorporation of cartilage adhering moieties, such as cationic molecules, can be used to enhance cartilage binding and lubrication properties of HA.

## Abstract 2

Hyaluronic acid (HA)-based lubricant functionalized with cationic branched poly-l-lysine molecules adhere to and lubricate cartilage.

## Graphical abstract

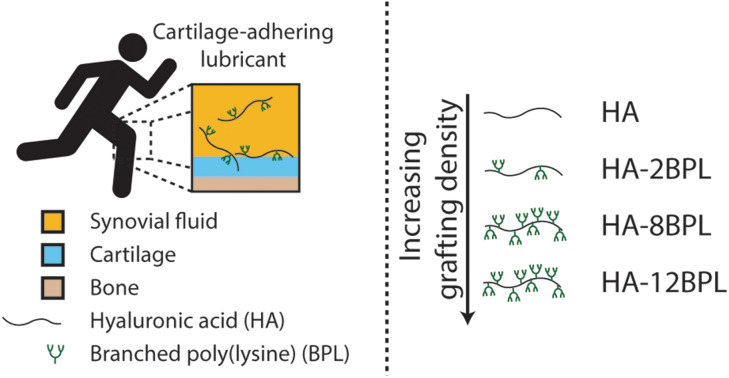

## Introduction

Joint lubrication plays an important role in cartilage health and function. Synovial fluid, the native lubricant in articular joints, is a complex mixture of various molecules such as hyaluronic acid (HA), lubricin (PRG4), phospholipids, and proteoglycans. These molecules synergistically play a protective role for the cartilage by providing the necessary lubrication of the sliding surfaces.^1,2^ HA, lubricin, and phospholipids, interact with both the cartilage and each other to create a hydrated dynamic network at the interface.^3,4^ These molecules together provide various modes of lubrication and thereby reduce friction experienced by the cartilage during various gaits and loading conditions.^1,5^

Osteoarthritis (OA) is a degenerative joint disease characterized by pain and alterations in both cartilage tissue, synovial fluid, and the lubrication ability of the joint.^6–10^ Current treatments for OA primarily focus on pain management *via* non-steroidal anti-inflammatory drugs (NSAIDs), corticosteroids, or viscosupplements to delay the time until a total knee replacement is needed.^11–15^ Hyaluronic acid-based viscosupplements have been an FDA-approved clinical intervention since 1997, and are thought to augment the properties of compromised synovial fluid and thereby addressing complications associated with cartilage degeneration.^7,11–13,16–18^ Currently approved HA viscosuplements are high molecular weight HA with occasional chemical crosslinking to improve its viscoelastic properties and joint retention time.^1,19^ While HA-based viscosupplements are extensively used in clinic, the therapeutic efficacy of HA-based viscosupplements is debated with most of them providing pain relief.^1,11,19^

High molecular weight HA, a natural component of synovial fluid, provides effective lubrication, and interacts with cartilage through other components of the synovial fluid such as lubricin.^1,6,20–23^ Lubricating molecules that are localized to the cartilage surface are necessary for boundary mode lubrication, a regime of lubrication which is characterized by many points of cartilage on cartilage contact, high normal loads on cartilage, and low synovial fluid viscosities.^1,5,24^ Coefficients of friction (COFs) on the cartilage are highest during boundary mode conditions, and *in vitro* lubrication studies have shown that synovial fluid can reduce coefficients of friction by as much as 150%.^25^ Most of the engineered lubricants are primarily focused on supplementing the viscoelastic properties of the synovial fluid and/or improving the retention of the polymers in the knee joint.^1,26,27^ However, a number of studies have designed lubricants to promote boundary mode lubrication and to improve their binding to cartilage.^28–31^ This includes incorporation of motifs that bind to extracellular matrix (ECM) components present on the cartilage surface or chemical modifications that enable direct conjugation of lubricants to the cartilage.^28,31–36^ Given the importance of lubricin in lubrication, lubricin-mimetic molecules have been developed and utilized to improve both cartilage binding and boundary lubrication.^1,24,37,38^ Since the cartilage ECM is composed of highly negatively charged proteoglycans, polymers with cationic moieties have been used to improve the cartilage binding properties and even to achieve boundary lubrication.^24,30^

**Fig. 1:**
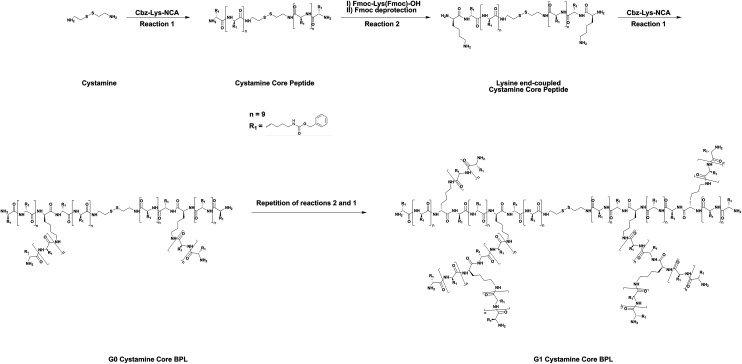
Synthesis of two headed BPL molecules.

We report the synthesis and development of a cartilage-binding HA-based lubricant, where the HA is sparsely encoded with cationic, branched poly(lysine) (BPL) peptides (Fig. 1a). In a previous study, we showed that BPL molecules with positive charge can rapidly adhere to and penetrate cartilage.^39^ Here, we use cationic BPL molecules to improve the cartilage binding properties of HA *via* electrostatic interactions. Towards this end, we conjugated BPL molecules onto the HA backbone—termed as HA-BPL. We varied the number of BPL molecules on the HA polymer chain to tune both the electrostatic interactions and cartilage binding. These HA-BPL molecules adhered to both healthy and OA cartilage tissues, including human tissues. The lubrication studies showed that HA-BPL molecules significantly reduced the coefficient of friction of articular cartilage under high-loading conditions.

## Experimental

### Materials

Hyaluronic acid of molecular weight 1 MDa (HA; Cat no. HA 1M) was purchased from Lifecore Biomedical. Hexane (Cat. no. 197360025) and triethanolamine (Cat. no. 139560010) were obtained from Acros Organics. Trifluoroacetic acid (TFA, Cat. no. A12198-36) and tris(2-carboxyethyl)phosphine hydrochloride (Cat. no. J60316-06) were obtained from Alfa Aesar. Triphosgene (Cat. no. T1467) was obtained from TCI Chemicals. *N*^ε^-Benzyloxycarbonyl-l-lysine (Cbz-Lys; Cat. no. 96840), tetrahydrofuran (THF; Cat. no. 186562), dimethlylformamide (DMF; Cat. no. 9227056), cystamine dihydrochloride (Cat. no. C121509), *N*^α^,*N*^ε^-di(9-fluorenylmethoxycarbonyl) l-lysine (*N*^α^,*N*^ε^-diFmoc-Lys; Cat. no. 8520410025), 2-(1*H*-benzo-triazole-1-yl)-oxy-1,1,3,3-tetramethyluronium hexafluorophosphate (HBTU; Cat. no. 8510060025), 1-hydroxybenzotriazole (HOBt; Cat. no. 157260), *N*,*N*-diisopropylethylamine (DIPEA; Cat. no. 496219), piperidine (Cat. no. 104094), hydrobromic acid (Cat. no. 18735), acetic acid (Cat. no. 695092), diethyl ether (Cat. no. 32203), methacrylic anhydride (Cat. no. 276685), ascorbic acid (Cat. no. A4544) and l-proline (Cat. no. P5607) were purchased from Sigma Aldrich. Dulbecco's modified Eagle's medium (DMEM; Cat. no. 11965092), HEPES (Cat. no. 15630080), sodium pyruvate (Cat. no. 11360070), MEM non-essential amino acids (MEM NEAA; Cat. no. 11140050), penicillin–streptomycin (Cat. no. 15140122), and Fetal Bovine Serum (FBS; Cat. no. 16000044) were obtained from Gibco. Live/dead assay kit was obtained from Invitrogen (Cat. no. L3224).

### Synthesis of BPL

Two-headed BPL molecules with a disulfide linker were synthesized using ring-opening polymerization (ROP) of *N*^ε^-benzyloxycarbonyl-l-lysine-*N*-carboxyanhydrides (Cbz-Lys-NCAs) as a monomer and 2,2′-dithiobis ethanamine as initiator.^39,40^ The details of the reaction steps are as follows:

#### Synthesis of *N*^ε^-benzyloxycarbonyl-l-lysine-*N*-carboxyanhydrides (Cbz-Lys-NCA)

*N* ^ε^-Benzyloxycarbonyl-l-lysine (Cbz-Lys, 3 equivalents) was reacted with triphosgene (1 equivalent) in anhydrous tetrahydrofuran at 50 °C for 3 hours. The reaction mixture was precipitated with excess hexane and washed repeatedly (at least 3 times) using hexane. The precipitate was then vacuum dried at 50 °C overnight to obtain Cbz-Lys-NCA as a white solid.

#### Synthesis of oligo(Cbz-l-lysine) with disulfide functional group

Cbz-Lys-NCA (20 equivalents) was dissolved in dry *N*,*N*-dimethylformamide (DMF, 100 mg mL^−1^) in a round bottomed flask fitted with a drying tube. 2,2′-Dithiobis ethanamine (1 equivalent), a disulfide group containing diamine initiator, was added to the reaction mixture and the reaction was continued at room temperature for about 5 days under continuous stirring. Following the reaction, the reaction mixture was added to ∼20-fold excess of cold water to precipitate the oligo(Cbz-l-lysine) core peptide. The precipitate was filtered, washed with water (≥3 times), and freeze-dried.

#### End coupling of *N*^α^,*N*^ε^-di(9-fluorenylmethoxycarbonyl-l-lysine) to the oligo(Cbz-l-lysine) core peptide

A solution of the oligo(Cbz-l-lysine) peptide in DMF was reacted with *N*^α^,*N*^ε^-di(9-fluorenylmethoxycarbonyl-l-lysine) (*N*^α^,*N*^ε^-diFmoc-Lys, 4 equivalents) using 2-(1*H*-benzo-triazole-1-yl)-oxy-1,1,3,3-tetramethyluronium hexafluorophosphate (HBTU, 4 equivalents), and 1-hydroxybenzotriazole (HOBt, 12 equivalents) and *N*,*N*-diisopropylethylamine (DIPEA, 10 equivalents) at room temperature for 3 days. The product was precipitated in excess of cold water, filtered, washed three times with water, and freeze-dried. The freeze-dried powder was washed 4 times with diethyl ether, and vacuum-dried to obtain *N*^α^,*N*^ε^-diFmoc-Lys conjugated core peptide.

#### Selective removal of Fmoc groups of the *N*^α^,*N*^ε^-diFmoc-Lys end-functionalized core peptide

A solution of the *N*^α^,*N*^ε^-diFmoc-Lys end-functionalized core peptide in DMF (∼200 mg mL^−1^) was reacted with 20% (v/v) piperidine under continuous stirring for an hour at room temperature and subsequently precipitated in water. The precipitate was filtered, washed repeatedly with water, and freeze-dried. The freeze-dried product was washed 4 times with diethyl ether, and vacuum-dried to obtain lysine end-functionalized core peptide.

#### Synthesis of branched BPL (G0 & G1) using lysine end-functionalized core peptide

The lysine end-functionalized core peptide was subsequently used as initiator along with the Cbz-Lys-NCA monomer to obtain next generation branched peptides. Higher generation of the branched peptides (G0 and G1) were then obtained by repeating the above steps of ring-opening polymerization, end functionalization and subsequent removal of Fmoc protecting groups. The degree of polymerization was controlled by maintaining the ratio of the Cbz-Lys-NCA monomer to the free amine groups of lysine (10 equivalent Cbz-Lys-NCA per 1 equivalent of free amine group of oligopeptides).

#### Cbz deprotection of branched BPL molecules

The Cbz protecting group was completely removed from BPL molecules to generate free amine groups in BPL molecules. Briefly, Cbz protected BPL was dissolved in trifluoroacetic acid (TFA, ∼100 mg per 3 mL) in a round-bottom flask. Then, a 4-fold molar excess of a 33 wt% solution of hydrobromic acid in acetic acid was added, and the reaction mixture was stirred for 1 hour at room temperature. The reaction mixture was then precipitated in excess of diethyl ether, and the product was isolated after filtration and vacuum-drying. Quantification of the product suggested >90% yield. Next, the product, fully deprotected two-headed BPL, was dissolved in water and the pH was adjusted to ∼7.0–7.5. The BPL solution was then dialyzed for 3–4 days by using a dialysis membrane with a molecular weight cut off 2 kDa against water. The solution was freeze-dried and the product, BPL molecules with free amine groups, was stored at −20 °C.

### Synthesis of HA-MA

Methacrylate groups were introduced into the HA polymer back bone by reacting HA with methacrylic anhydride.^41^ Briefly, HA (1 equivalent with respect to dimeric repeat unit) was dissolved in deionized (DI) water. Methacrylic anhydride was added to the HA solution (1 equivalent), and the pH of the reaction mixture was adjusted to 8–8.5 by adding 5 N NaOH. The reaction was continued for ∼2 hours at room temperature with constant maintenance of the pH. The reaction mixture was then dialyzed using DI water for 3–4 days, and the solution was freeze dried to obtain the methacrylated HA (HA-MA).

### Conjugation of BPL to HA-MA

BPL conjugation to HA-MA was performed by first reducing the disulfide group containing two-headed BPL molecules to a functional thiol group containing a single-headed BPL. These single-headed BPL molecules were then reacted with HA-MA through a Michael addition reaction. The amount of BPL molecules was (2–12 equivalents per 2500 HA-MA dimeric repeat unit) varied in the reaction mixture to generate modified HA with varying numbers of BPL molecules. Briefly, two-headed BPL molecules (2 equivalents, 8 equivalents, or 12 equivalents per 2500 HA-MA dimeric repeat unit) were dissolved in water and to this tris(2-carboxyethyl)phosphine hydrochloride (TCEP, 2 equivalents with respect to two-headed BPL) was added and reacted for 6 hours at room temperature. Next, the solution containing reduced BPL was added to a solution of HA-MA (1 equivalent) in triethanolamine buffer at pH 8.0 (5 mg mL^−1^). The reaction mixture was reacted for 36 hours under continuous stirring at room temperature, dialyzed for 3–4 days against DI water (molecular weight cutoff 12–14 kDa membrane), and freeze-dried. The product, BPL conjugated hyaluronic acid (HA-BPL), was stored at −20 °C. The resulting HA-BPL molecules termed hereafter as HA-2BPL, HA-8BPL, and HA-12BPL.

### Characterization of BPL

#### Proton nuclear magnetic resonance spectroscopy (^1^H NMR)

BPL and HA-BPL molecules were characterized by proton nuclear magnetic resonance spectroscopy (^1^H NMR). ^1^H NMR measurements were carried out using deuterated water or dimethyl sulfoxide-d_6_ at a concentration of ∼1 wt%. The spectra were recorded with a 500 MHz Varian VNMRS spectrometer at room temperature.

#### Rheological measurements

The viscoelastic properties of HA and HA-BPL molecules were characterized by frequency and strain sweep measurements using an AR G2 rheometer (TA Instruments) with a parallel plate geometry (8 mm diameter) as previously described.^26^ Prior to the measurements, the polymers were dissolved in PBS at a concentration of 5 wt%. To determine the onset of the non-linear regime, we performed strain sweep measurements from 0.1% to 10% strain at a frequency of 1 Hz. Next, we used frequency sweeps from 0.1 Hz to 10 Hz at 1% strain to determine the frequency dependent storage (*G*′) and loss (*G*′′) moduli of the HA and HA-BPL polymer solutions. Additionally, we determined the shear thinning behavior the HA and HA-BPL, by measuring the viscosity as a function of shear rate (0.1 s^−1^ to 10 s^−1^).

#### Optical density measurements

To examine whether the incorporation of cationic BPL molecules leads to aggregation of the polymer chains, we carried out optical density measurements of HA and HA-BPL molecules of varying concentration and BPL grafting density. The optical density measurements were carried out at a wavelength of 600 nm using a Tecan Infinite F200 pro fluorescence plate reader. HA and HA-BPLs were dissolved in PBS (pH 7.4) at varying concentration (0.25–12.0 mg mL^−1^). Approximately, 200 μL of each polymer solution was used in a Costar 96-well flat black plate to measure the optical density.

### *In vitro* cartilage experiments

To determine the BPL grafting dependent adhesion to cartilage, we used healthy and OA mimetic bovine cartilage explants, which were generated as described earlier,^39^ and human OA cartilage tissues as described below.

#### Bovine cartilage isolation

Cartilage explants were isolated from 2 week-old bovine knee joints obtained from Research 87 (Boylston, MA). Cartilage plugs of 4 mm in diameter and 4 mm in height were isolated from the femoral condyles of the cartilage and pooled for cytotoxicity and adhesion experiments. Cartilage plugs (8 mm in diameter and 4–6 mm in height) were isolated from the medial and lateral facets of the trochlear groove and pooled for lubrication experiments. All cartilage explants were cultured in 1 mL of chondrocyte media in a 24-well plate and the media was changed every other day. Chondrocyte media was prepared by mixing 10% (v/v) FBS, 1% (v/v) ascorbic acid, 1% (v/v) HEPES, 1% (v/v) l-proline, 1% (v/v) sodium pyruvate, 1% (v/v) MEM non-essential amino acids solution, and 1% (v/v) penicillin–streptomycin, to DMEM with high glucose. The solution was filtered through a vacuum filter flask with a 0.22 μm filter and stored at 4 °C until use.

#### Cytotoxicity/viability assay after culturing bovine cartilage explants with HA-BPL

Bovine cartilage explants were incubated in chondrocyte medium with HA, HA-2BPL and HA-8BPL (1.5 wt%). The explants were incubated for 24 hours at 37 °C and 5% CO_2_. After incubation, the cartilage explants were cut into sections of 100–200 μm and incubated with 500 μL of chondrocyte medium with calcein AM and ethidium homodimer-1 at concentrations of 2 μM and 4 μM, respectively. After 30 minutes of incubation, the cartilage explants were washed with PBS and imaged with a Keyence BZ-X710 fluorescence microscope. Fluorescence images were quantified to determine the live (green) and dead (red) cells.^42^

#### Assessing cartilage adhesion of HA and modified HA using cartilage explants

Cartilage explants (4 mm in diameter and 4 mm height) were equilibrated in chondrocyte media for 24 hours prior to use. A custom designed PDMS device was used for the experiments that enabled selective exposure of the superficial zone of the cartilage to the HA and HA-BPL molecules^39^ as shown schematically in Fig. S13.† The device was placed in a 24-well plate containing 500 μL of PBS to maintain humidity and limit dehydration of the tissue and evaporation of the HA and HA-BPL solutions. The device used in this experiment consisted of a hollow cylinder (15 mm height × 12 mm diameter) with an upper, middle and lower compartment with diameters of 10 mm, 3.95 mm and 2 mm, respectively. The upper compartment was designed to house HA and HA-BPL solutions, the middle compartment housed the cartilage explants, and the lower compartment to allow the cartilage explant to access the PBS, to avoid drying of the tissue. For the adsorption experiments, cartilage explants were press fitted into the middle chamber of the device. Fluorescently labelled HA and HA-BPL molecules were dissolved in PBS at concentrations of 5 mg mL^−1^. 200 μL of the fluorescently labelled solution was placed in the upper compartment such that the surface of the cartilage explant was exposed to the solution as in a synovial joint. The cultures were incubated at 37 °C for various durations of time—30 minutes, 6 hours, and 24 hours. After exposure to the polymers, the cartilage explants were washed with PBS, fixed with 4% PFA, dehydrated in sucrose, embedded in OCT sectioning medium, sectioned into 15 μm thick sections, mounted using a DAPI mounting medium, and imaged with a Keyence BZ-X710 fluorescence microscope to qualitatively characterize the adhesion of the polymers by analyzing its presence in the superficial zone of the cartilage (upper 10% of the cartilage surface) into the full-thickness cartilage explants.^43^

#### Generation of OA-mimetic cartilage by cytokine challenging

To create OA-mimetic cartilage, the bovine explants were exposed to IL-1β as described elsewhere.^39^ Briefly, the cartilage explants were cultured in chondrocyte medium containing IL-1β (Cat. no. 201-LB-005; R&D Systems) at 25 ng mL^−1^ for 6 days.

#### Human cartilage tissue isolation

Human osteoarthritic cartilage specimens were collected from individuals undergoing total knee arthroplasty at Duke University, Department of Orthopedic Surgery with approval by the Institutional Review Board (IRB number: Pro00115320). From the least affected sections, cartilage plugs of 4 mm diameter and ∼2 mm height were punched out using a biopsy punch. The fresh cartilage plugs were equilibrated at 37 °C and 5% CO_2_ in chondrocyte media in a 24-well plate for 24 hours and used for the adhesion studies.

#### Shear force assay

We fabricated a perfusable, microfluidic device for a shear force assay. To this end, we used photolithography to pattern designs of 36 mm × 5.6 mm on a silicon wafer using SU-8100 sacrificial photoresist. Briefly, the photoresist was spin coated on a cleaned wafer at 3000 rpm for 30 seconds. Next, the wafer was baked for 10 minutes first at 65 °C and then at 95 °C for 30 minutes. After this step, the wafer was exposed to 365 nm wavelength light through a photomask and baked at 65 °C for 1 minute followed by 95 °C for 10 minutes. Finally, the wafer was rinsed with SU-8 developer to remove any undeveloped photoresist, and the resulting master mold was cleaned with isopropanol followed by water and stored until use. Polydimethylsiloxane (PDMS) solution was mixed at a ratio of 10 : 1, base to crosslinker, poured into the master mold, and kept at 60 °C for 2 hours to cure. After curing, the PDMS was cut into rectangular pieces. A 1 mm biopsy punch was used to punch out inlet and outlet holes, and a 4 mm biopsy punch was used to punch a hole in the center of the chamber to house the cartilage explant.

Surfaces of the cartilage explants were exposed to Cy5 -tagged HA and HA-BPL molecules for 30 minutes as described earlier. The cartilage explants were then transferred to excess PBS for 5 minutes to remove unbound polymers. The cartilage explants were then inserted into the device and secured with Eukitt Mounting Matrix. The inlet of the device was connected to a peristaltic pump to support PBS perfusion and the cartilage explants were exposed to shear flow at a flow rate of 50 μL per minute for 10 minutes. The fluid flow in the device was modeled using COMSOL before experiments were completed. The cartilage with the adhered polymer was imaged on a Keyence BZ-X710 fluorescence microscope before and after the exposure to the flow, and the fluorescence intensity was quantified to determine the amount of adhered polymer.

#### Coefficient of friction measurements

The coefficient of friction was measured using a TA-AR-G2 rheometer, with an 8 mm parallel plate (disk) geometry. Cartilage explants were harvested from bovine knee joints and allowed to equilibrate in chondrocyte medium before the experiments. After the initial equilibration, the cartilage explants were trimmed to get samples with heights (or thickness) of 2 mm and either 8 mm diameter or 4 mm diameter. The 8 mm diameter explants were used as the stationary substrate while the 4 mm diameter explant was glued to the rotating upper plate. To limit the contribution from the innate lubricin present in the cartilage to the lubrication, the cartilage explants were incubated in 1.5 M NaCl at 37 °C, to remove any surface bound lubricin, and washed in PBS with protease inhibitors for an hour.^27^ Next, the explants were incubated for 24 hours either in a solution of PBS, or 3 wt% HA or HA-BPL at 4 °C, and washed with PBS for 30 minutes to remove any unbound HA or HA-BPL molecules. These cartilage explants were glued to a sandpaper (3 M; 120 grit) which was then glued to the plates with cyanoacrylate adhesive (Super Glue Corporation) to ensure close contact with the cartilage. Prior to the friction tests, the sandwiched cartilage samples were brought into contact with a normal force of 0.01N (establishing contact), and then compressed to 30% strain. To improve the reproducibility and limit contributions from the confounding factors, the cartilage samples were sheared against each other by a 360° rotation clockwise, followed by a 360° rotation counterclockwise, and followed by 1 hour at the fixed strain for stress relaxation. After this initialization, the friction test of the cartilage samples consisted of three rotations (clockwise, counterclockwise, and again clockwise) at an effective sliding velocity of 0.1 mm s^−1^ for 2.5 minutes. This sliding velocity was approximated as the angular velocity times the radius of the upper cartilage sample. We determined the coefficient of friction (COF) in the cartilage-on-cartilage interface using the equation:^44^*μ* = (4 × *τ*)/(3 × *R* × *F*_n_)where *μ* is the coefficient of friction, *τ* is the torque, *R* is the radius and *F*_n_ is the normal force. The coefficient of friction during the last two rotation measurements were averaged and reported.

## Results and discussion

### Synthesis and characterization of HA-BPL molecules

**Fig. 2:**
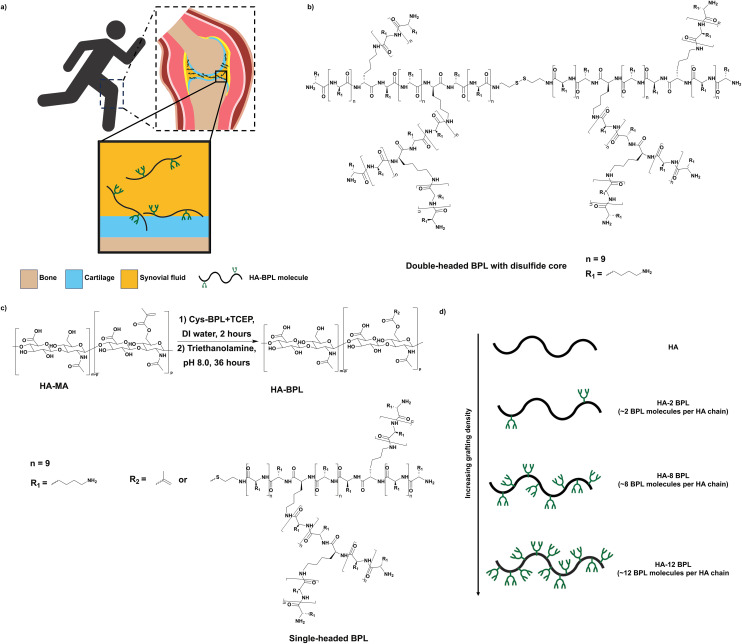
Molecular engineering of HA-BPL molecules. (a) HA-BPL molecules adhere to cartilage surface. (b) Two-headed BPL molecule. (c) Conjugation of BPL molecules to HA. (d) Design of HA-BPL molecules (HA and BPL molecules are not drawn to scale in the schematic representations).

HA-BPL polymers were synthesized *via* a Michael addition reaction between the terminal thiol groups of the thiolated BPL molecules and methacrylated HA (Fig. 1 and 2). The BPL molecules were synthesized by ring-opening polymerization (ROP) reactions using *N*^ε^-benzyloxycarbonyl-l-lysine *N*-carboxyanhydride (Cbz-Lys-NCA) as a monomer and 2,2′-dithiobis ethanamine (DBE) as an initiator as described elsewhere (Fig. 1 and 2b).^39,40^ First, a lysine oligomer (core peptide) with a disulfide group at the center was generated by reacting DBE with Cbz-Lys-NCA. In a subsequent reaction, the core peptide was end-functionalized with *N*^α^,*N*^ε^-di(9-fluorenylmethoxycarbonyl) l-lysine (*N*^α^,*N*^ε^-diFmoc-Lys) under standard amide coupling conditions.^39,40^ Finally, the Fmoc groups at the terminal l-lysine residues of the core peptide were removed by reacting with piperidine, which generated two new terminal primary amine groups on each side of the molecule with a disulfide center. These amine groups served as initiators for the addition of the next generation of branches. Repetition of the ring-opening polymerization, end functionalization, and deprotection sequence produced branched two-headed poly(l-lysine) peptides (Fig. 2b). A 10-fold molar excess of Cbz-Lys-NCA monomer with respect to each primary amine group was used throughout the ring-opening polymerization process. Successful completion of each of the reaction steps and intermediate products was confirmed by ^1^H NMR.

Assuming that each of the *N*^α^,*N*^ε^-diFmoc-Lys couplings and subsequent Fmoc deprotections proceeds without loss, the theoretical number of terminal lysine moieties in the BPL molecules, the average number of lysine repeating units per branch (DP_branch_) and the number average degree of polymerization (DP_total_) for the BPL molecules were estimated and are shown in Table S1.† The theoretical values were then verified by the ^1^H NMR spectra analyses (Fig. S1–S3†). Specifically, the comparison of protons from the methine groups of terminal lysine moieties with those of the residual methine protons of other lysine moieties quantifies the DP_total_ and DP_branch_ for the BPL molecules (Fig. S1–S3 and Table S2†). The DP_branch_ and DP_total_ values obtained *via*^1^H NMR show minimal deviation from the theoretically calculated values suggesting the absence of major structural imperfections or side-chain reactions.

Next, HA (MW ∼ 1 MDa) was methacrylated by reacting with methacrylic anhydride as described elsewhere (Fig. S4†).^41^ Methacrylation of HA was confirmed from the ^1^H NMR spectrum which showed presence of peaks at 5.8 and 6.2 ppm corresponding to the methylene protons of the methacrylate groups (Fig. S5†). The degree of methacrylation was estimated to be 2 ± 0.5% per dimeric repeat unit. Finally, incorporation of BPL molecules to the HA polymer chain was achieved *via* the Michael addition reaction where the methacrylate groups of the HA-MA were reacted with the thiol groups of thiol-terminated BPL molecules (Fig. 2c). The thiol-terminated BPL molecules were obtained *via in situ* reduction of the disulfide bond of the BPL molecules (Fig. S6†). Successful incorporation of BPL molecules to the HA polymer chain was confirmed *via*^1^H NMR spectrum, which showed the presence of methylene protons of lysine unit at 1.43–1.5 ppm, 1.7–1.8 ppm, and 2.03 ppm, respectively, in addition to the peaks at 3.2–3.8 ppm corresponding to the C2–C6 protons and 4.2–4.6 ppm C1 protons of HA (Fig. S7–S9†). The ratio of the area under the curve for methylene protons of the lysine unit to the area under the curve for the C1 proton of HA was used to calculate the degree of BPL conjugation. The molar ratio of BPL to HA was adjusted during the coupling reaction to achieve HA-BPL molecules with varying BPL grafting densities, ranging from 2–12 BPL molecules per HA chain (*i.e.*, 2–12 BPL molecules per 2500 HA dimeric repeat units) (Fig. 2d). The presence of the disulfide functional group at the center of the two-headed BPL molecules allowed selective cleavage of the two-headed molecules to generate BPL molecules with terminal thiol groups, which in turn allowed controlled, *in situ* chemical conjugation of the BPL to the HA backbone.

### Effect of BPL grafting density on the physical properties of the HA molecule

**Fig. 3:**
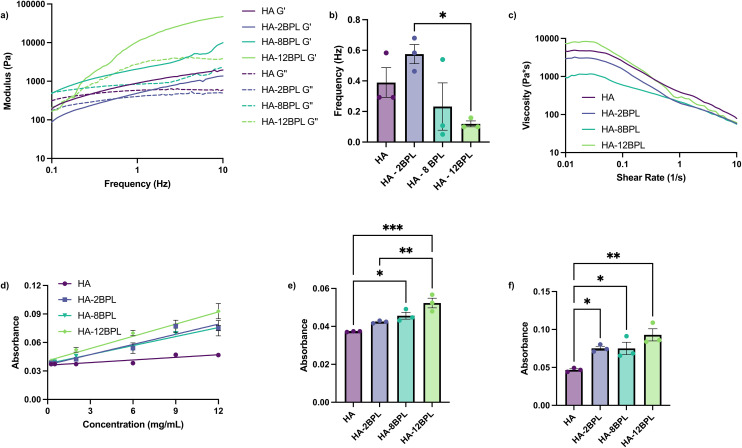
Physical characterization of HA and HA-BPL molecules. (a) Frequency sweep, (b) crossover frequency, (c) shear rate sweep, (d) optical density at 600 nm from concentrations of 0.25 mg mL^−1^ to 12 mg mL^−1^, (e) optical density at 600 nm at 2 mg mL^−1^, and (f) optical density at 600 nm at 12 mg mL^−1^.

To study the rheological effect of BPL grafting on the HA backbones, we measured the viscoelastic properties of HA-BPL solutions at different BPL grafting densities and compared these measurements to those obtained with unmodified HA controls. To this end, we employed oscillatory rheometry and performed strain sweeps on the HA-BPL solutions at a frequency of 1 Hz from 0.1% strain to 10% strain to identify the linear region. The linear region was determined by strains at which no significant changes of storage modulus (*G*′) or loss modulus (*G*′′) were observed (Fig. S10†). Subsequent frequency sweep measurements (0.1–10 Hz) of HA-BPL solutions in the linear regime revealed that the grafting density of BPL molecules had an influence on the rheological properties of the HA molecules in solution, albeit with subtle changes. For instance, HA-2BPL solutions (*i.e.*, 2 BPL molecules per HA polymer chain) had lower dynamic moduli (*G*′ and *G*′′) compared to the corresponding unmodified HA (Fig. 3a). However, the *G*′ and *G*′′ values increased with further increase in the number of BPL molecules on the HA polymer chain. For example, HA-8BPL and HA-12BPL solutions had higher storage and loss moduli than HA-2BPL, and the *G*′ and *G*′′ values of these HA-BPL solutions were similar to those of the unmodified HA molecules (Fig. 3a). We attribute the decrease in the dynamic moduli for the HA-2BPL solutions compared to those of the unmodified HA to the presence of the bulky BPL molecules that prevent the polymer chains from coming together. The increase in moduli with increasing number of BPL molecules (*i.e.*, 8 and 12 BPL molecules per HA chain), is likely due to the increased physical interactions between the positively charged BPL molecules and the anionic HA molecules. Consistent with these findings, the BPL grafting density also showed a similar trend on the crossover frequency, *i.e.*, the frequency at which *G*′ > *G*′′ in frequency sweep measurements (Fig. 3b). The higher crossover frequency for HA-2BPL solutions suggests that relatively fewer intermolecular interactions occurred compared to unmodified HA. We surmise that the observed decrease in crossover frequency with further increasing grafting density (*e.g.*, HA-8BPL and HA-12BPL) is due to increased intermolecular interactions between the polymer chains. Finally, the shear rate sweep measurements showed shear thinning behavior for all the HA-BPL solutions, similar to that of the HA solution (Fig. 3c).

Since intermolecular interactions between the chains can lead to aggregation, we carried out optical density (OD) measurements at 600 nm for HA and HA-BPL molecules with varying concentrations. The optical density of the unmodified HA showed a constant value for all the tested concentrations (0.25 mg mL^−1^–12 mg mL^−1^) (Fig. 3d). In contrast, the HA-BPL polymers displayed a concentration and grafting density dependent turbidity (Fig. 3d). At lower concentrations (2 mg mL^−1^), absorbance of the HA-BPL polymers increased with increasing the number of BPL units in the polymer chain (Fig. 3e). Consistent with this observation, while higher optical density was observed in all HA-BPL molecules with increasing concentration, a significantly higher turbidity was observed for HA-BPL polymers especially at 12 mg mL^−1^ concentration (Fig. 3f). Because of the increased aggregation tendency of the HA-12BPL, modified HA with a lower number of BPL per polymer chain (HA-2BPL and HA-8BPL) were used for the subsequent studies.

### Cationic BPL molecules enhanced cartilage adhesion of the HA polymers

**Fig. 4:**
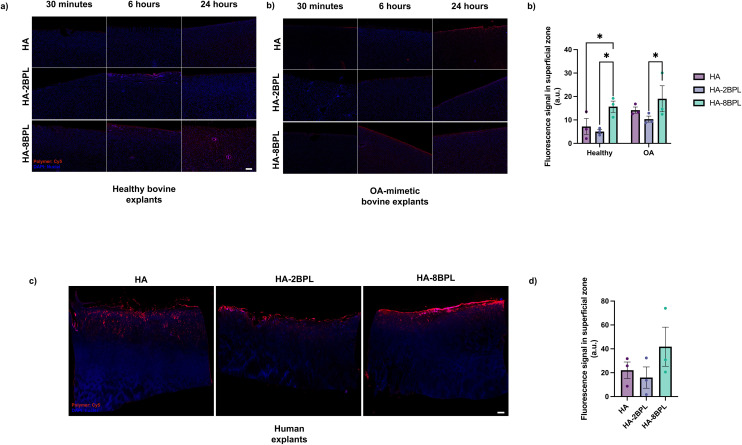
Adhesion of HA and HA-BPL molecules to cartilage. (a) Adhesion to healthy and OA-mimetic bovine cartilage explants (scale bar = 400 μm). (b) Quantification of adhesion to superficial zone of bovine cartilage explants by 24 hours. (c) Adhesion to osteoarthritic human cartilage explants at 6 hours (scale bar = 400 μm). (d) Quantification of HA and HA-BPL molecules present in the cartilage surface at 6 hours.

We next examined the effect of BPL conjugation on the cartilage adhesion properties of HA by using both healthy and OA-mimetic cartilage explants. The OA-mimetic explants were used to determine the effect of proteoglycan loss on the adhesion of HA-derived molecules. The findings were extended to human tissues by using human OA cartilage explants. The adhesion of the HA and HA-BPL molecules were assessed by using cyanine-5 conjugated molecules. The HA and HA-BPL molecules were delivered to the cartilage explant surface as a function of time using a custom device designed for selective exposure of the HA molecules to the cartilage surface. Images of the cartilage explants following their exposure to fluorescently labelled HA and HA-BPL molecules suggest that all the HA-BPL molecules adhered to the cartilage surface by 30 minutes (the earliest time point measured), and remained adhered to the surface for at least 24 hours (the latest time point measured) for both healthy and OA-mimetic bovine cartilage explants, albeit with subtle differences (Fig. 4a–c). The time dependent analyses showed an increase in fluorescence intensity in cartilage explants exposed to HA and HA-BPL molecules at 6 hours which then plateaued by 24 hours; the trend was similar for both the healthy and OA-mimetic bovine explants (Fig. 4a–c and S12†). Between the healthy and OA mimetic explants, the latter group showed higher adhesion of HA and HA-BPL molecules to the cartilage surface (Table S3†). We attribute this to the proteoglycan loss in the OA-mimetic explants, which leads to a less dense cartilage network, thus making the cartilage more porous, and enabling the HA-molecules to penetrate into the cartilage. Among the HA-BPL molecules, HA-8BPL exhibited higher adhesion which is likely due to the higher positive surface charge which enables more binding sites with the cartilage surface *via* electrostatic interaction (Fig. 4a, b and S12†).^39^

As with the bovine explants, HA and HA-BPL molecules also adhered to the human cartilage explants, showing a similar trend. After 6 hours of incubation (the only experimental time point tested), both HA and HA-BPL molecules adhered and penetrated into the superficial zone of the cartilage explants (Fig. 4d and e). As suggested by the fluorescence intensity HA-8BPL molecules showed higher adhesion and localization while HA and HA-2BPL molecules showed similar levels of adhesion (Fig. 4d and e).

**Fig. 5:**
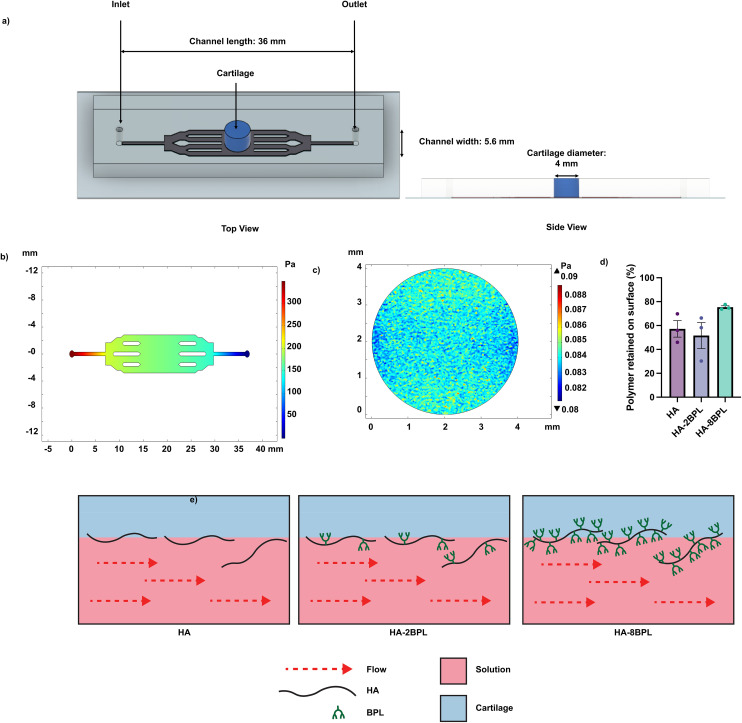
Adhesion strength assay using fluid shear flow. (a) Device used to create fluid shear flow on cartilage explants. (b) COMSOL simulation showing pressure in the device. (c) COMSOL simulation of shear force on the cartilage explant in the device. (d) Quantification of polymer remaining on the cartilage explant after fluid shear flow. (e) Schematic describing adhesion of the HA and HA-BPL molecules (HA and BPL molecules are not drawn to scale in schematic representations).

To evaluate HA-BPL molecule adhesion to cartilage, we conducted an adhesion strength assay. This involved first exposing the cartilage surface to HA or HA-BPL solutions, then applying a shear force, and measuring the amount of polymer retained on the cartilage surface (Fig. 5a). To determine the flow characteristics in the device COMSOL Multiphysics with Fluid Mechanics module was used to model the flow behavior. The COMSOL model indicated laminar flow and uniform pressure distribution on the cartilage explant surface. The fluid shear stress on the cartilage surface was calculated to be 0.083 Pa (Fig. 5b and c). Consistent with the adhesion studies discussed above, experimental shear force measurements showed that HA-8BPL molecules adhered to the cartilage were more resilient to the shear force applied and remained bound to the cartilage (Fig. 5d). We attribute the higher retention of the HA-8BPL molecules to multiple contact points generated by the positively charged BPL molecules (∼8 BPL molecules per polymer chain) along the cartilage tissue (Fig. 5e).

### HA-BPL molecules showed minimal toxicity

**Fig. 6:**
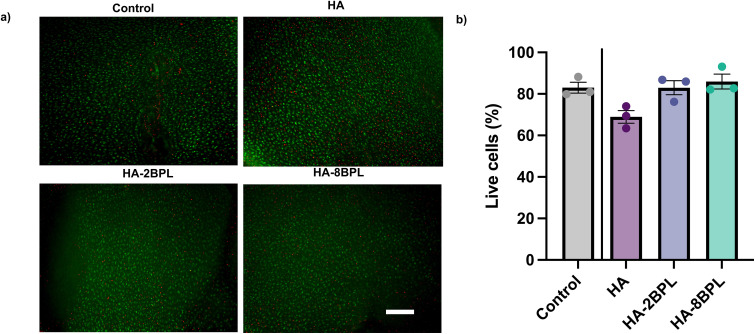
Cell compatibility in bovine cartilage explants after 24 hours of culture with HA and HA-BPL molecules. (a) Representative images of cell viability (live: green and red: dead, scale bar = 200 μm). (b) Quantification of cell viability.

To determine the cytocompatibility of the HA-BPL molecules, cartilage explants were incubated with HA, HA-2BPL, and HA-8BPL solutions and analysed for chondrocyte viability. No significant difference in cell viability was observed for any of the polymeric molecules (Fig. 6). Overall, these results indicate that the conjugation of BPL molecules does not contribute to cytotoxicity. This is not surprising given that only a small number of cationic BPL molecules are incorporated into the HA molecules.

### BPL grafting enhanced lubrication properties of HA

**Fig. 7:**
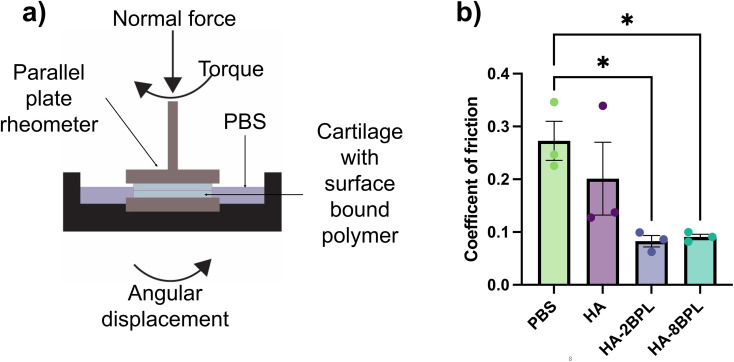
Lubrication in a cartilage-on-cartilage shear setup. (a) Schematic representation of parallel plate rheometer used to measure coefficient of friction. (b) Coefficient of friction of cartilage-on-cartilage shear setup after incubating with PBS, HA, or HA-BPL molecules.

We next examined the effect of BPL grafting on the lubrication function of the HA molecules. Prior studies have shown that lubricants that are adhered to the cartilage surface promote boundary mode lubrication, which involves significant cartilage-on-cartilage contact and high normal loads. We therefore investigated the lubrication properties of HA-BPL molecules in a cartilage-on-cartilage shear setup while imposing 30% strain to the cartilage tissue (Fig. 7a). Using an oscillatory rheometer, the coefficients of friction of bovine cartilage explants in the presence of PBS, HA, or HA-BPL solutions were determined. We observed a significant decrease in the coefficient of friction in samples lubricated with HA-2BPL and HA-8BPL solutions compared to cartilage samples lubricated with PBS or unmodified HA. Interestingly, both HA-2BPL and HA-8BPL molecules had similar coefficients of friction. The coefficient of friction when the cartilage surfaces were lubricated with HA-BPL solutions was reduced by ∼70% and 57% compared to PBS and HA controls, respectively, and there was no significant difference in the coefficient of friction between HA and PBS. The lower coefficient of friction in cartilage samples lubricated with HA-BPL solutions likely arises because of the increased interactions between the HA-BPL molecules and the cartilage explants (Fig. 7b).

## Conclusions

In summary, we designed and synthesized hyaluronic acid (HA) molecules encoded with cationic branched poly-l-lysine (BPL) molecules to enhance interactions with the cartilage surface. Through a controlled Michael addition reaction, we successfully conjugated 2–12 BPL molecules per HA polymer chain. Compared to HA alone, HA-BPL molecules adhered to the cartilage and formed a strong coating with minimal cytotoxicity. The HA-BPL molecules demonstrated a significant improvement in lubrication of cartilage explants under high strain in a cartilage-on-cartilage friction measurement. Together the results suggest that cationic, cartilage-binding BPL molecules present an exciting approach for functionalizing HA to improve its lubrication function. While this study focused solely on HA modification with cationic BPL molecules, this approach can be combined with other HA modifications, such as incorporating amphiphilic molecules known to enhance lubrication or conjugating anabolic biomolecules to promote cartilage regeneration.

## Ethical statement

All experiments involving human cartilage tissues were performed in accordance with the Guidelines of the Institutional Review Board (IRB number: Pro00115320), and experiments were approved by the ethics committee at Duke university.

## Data availability

The data supporting this article have been included as part of the ESI.† Raw data is available upon request from authors.

## Conflicts of interest

There are no conflicts to declare.

## Supplementary Material

BM-012-D4BM00532E-s001
