# Supplementary material for: Grafting of cationic molecules to hyaluronic acid improves adsorption and cartilage lubrication
Source: Biomater Sci. 2024 Aug 8;12(18):4747–58. doi: 10.1039/d4bm00532e (PMC11310657; doi:10.1039/d4bm00532e)
Supplement: BM-012-D4BM00532E-s001 [file BM-012-D4BM00532E-s001.pdf]

Supplementary Information (SI) for Biomaterials Science.
This journal is © The Royal Society of Chemistry 2024

Supplementary Information for

Grafting of Cationic Molecules to Hyaluronic Acid Improves Adsorption and Cartilage Lubrication

Gavin Gonzales[1], Jiaul Hoque[2], Colin Kaeo[1], Stefan Zauscher[3], and Shyni Varghese[1, 2, 3*]
[1]Department of Biomedical Engineering, Duke University, Durham, NC 27710, USA
[2]Department of Orthopaedic Surgery, Duke University School of Medicine, Durham, NC 27710, USA
[3]Department of Mechanical Engineering and Materials Science, Duke University, Durham, NC 27710, USA

*To whom correspondence should be addressed:

E-mail: shyni.varghese@duke.edu, Tel: +1-919-660-5273

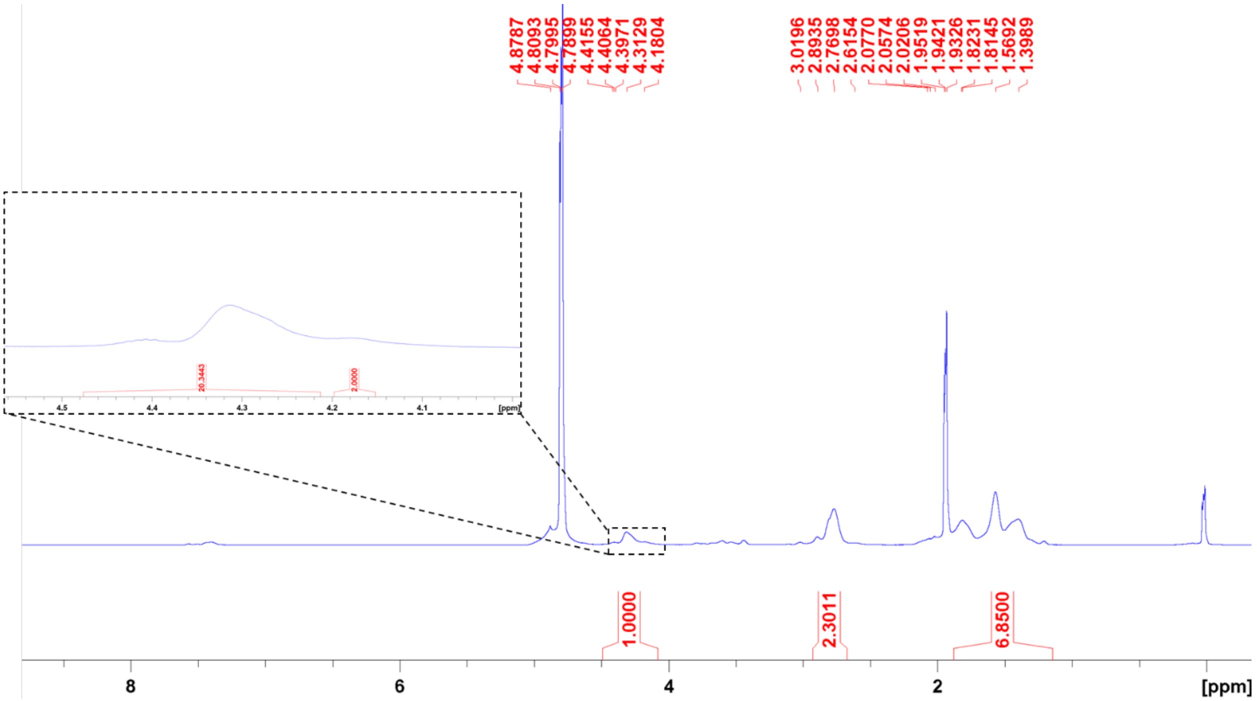

Figure S1. ^{1}HNMR Spectrum of fully deprotected core peptide recorded in D_2O at 25 °C.

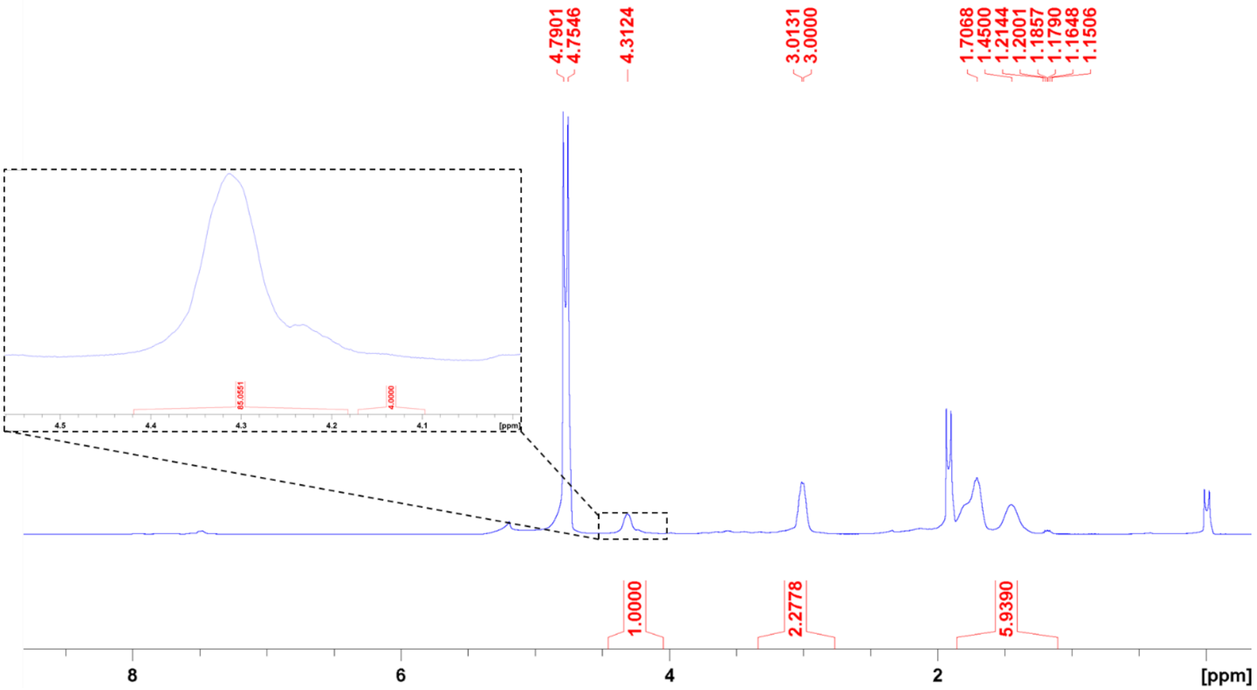

Figure S2. ^1HNMR Spectrum of fully deprotected G0 peptide recorded in D_2O at 25 °C.

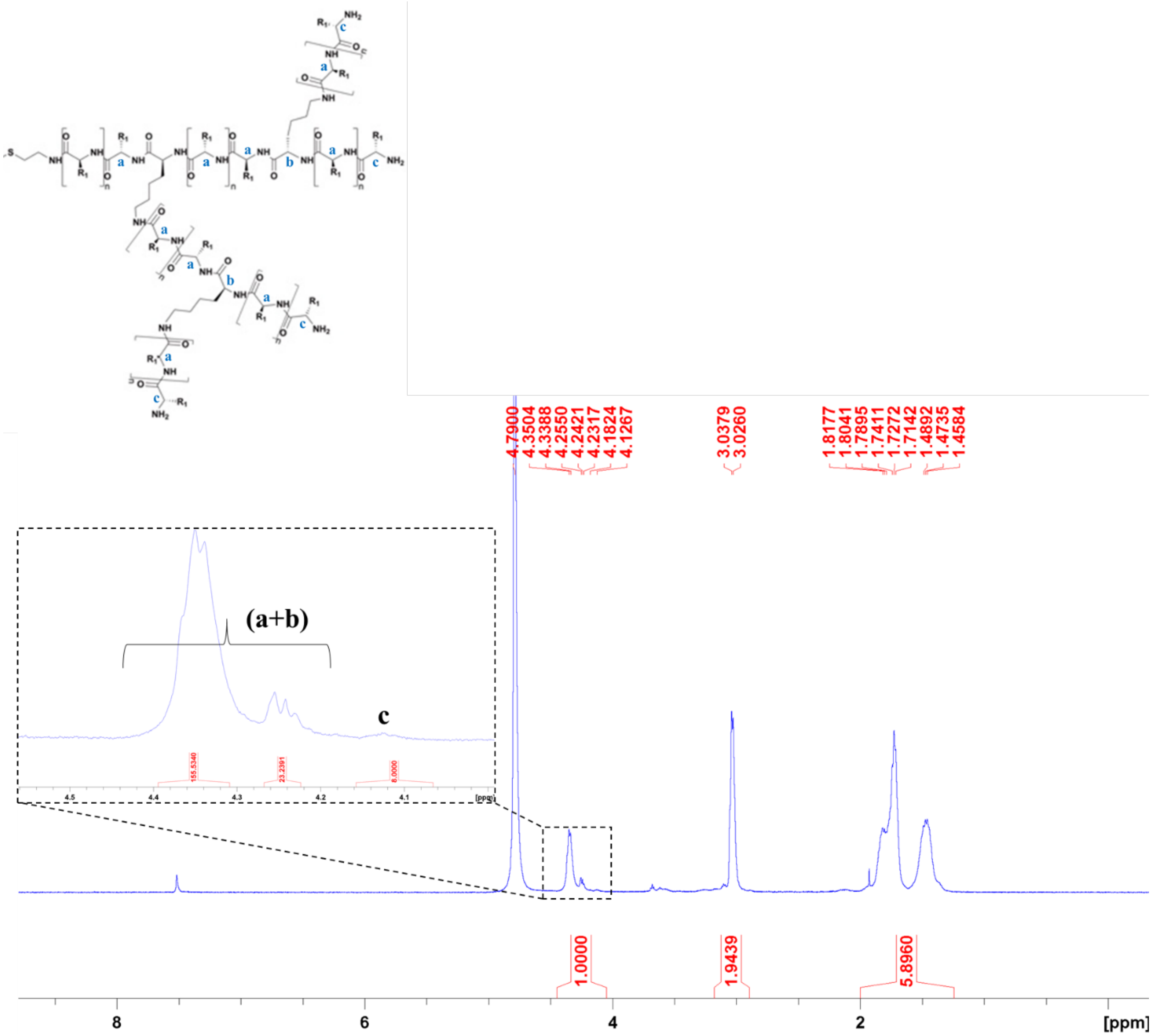

Figure S3. ^{1}HNMR Spectrum of fully deprotected G1 peptide recorded in D_2O at 25 °C.

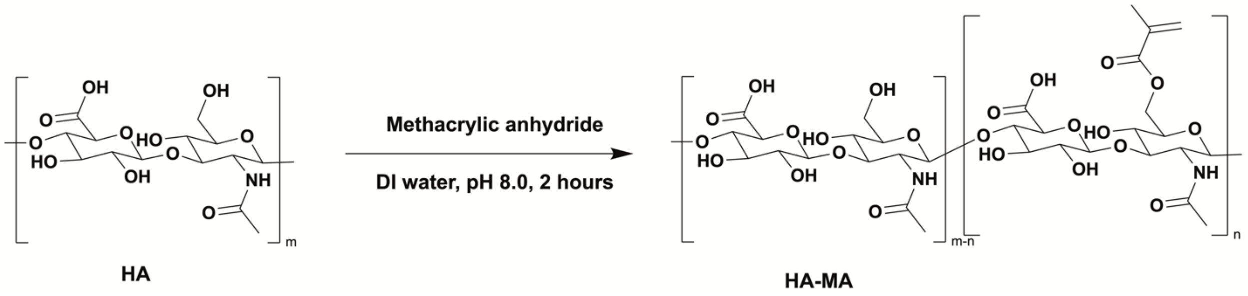

Figure S4. Synthesis of HA-MA from HA. Reaction scheme showing the incorporation of methacrylate groups in the HA by reacting HA with methacrylic anhydride.

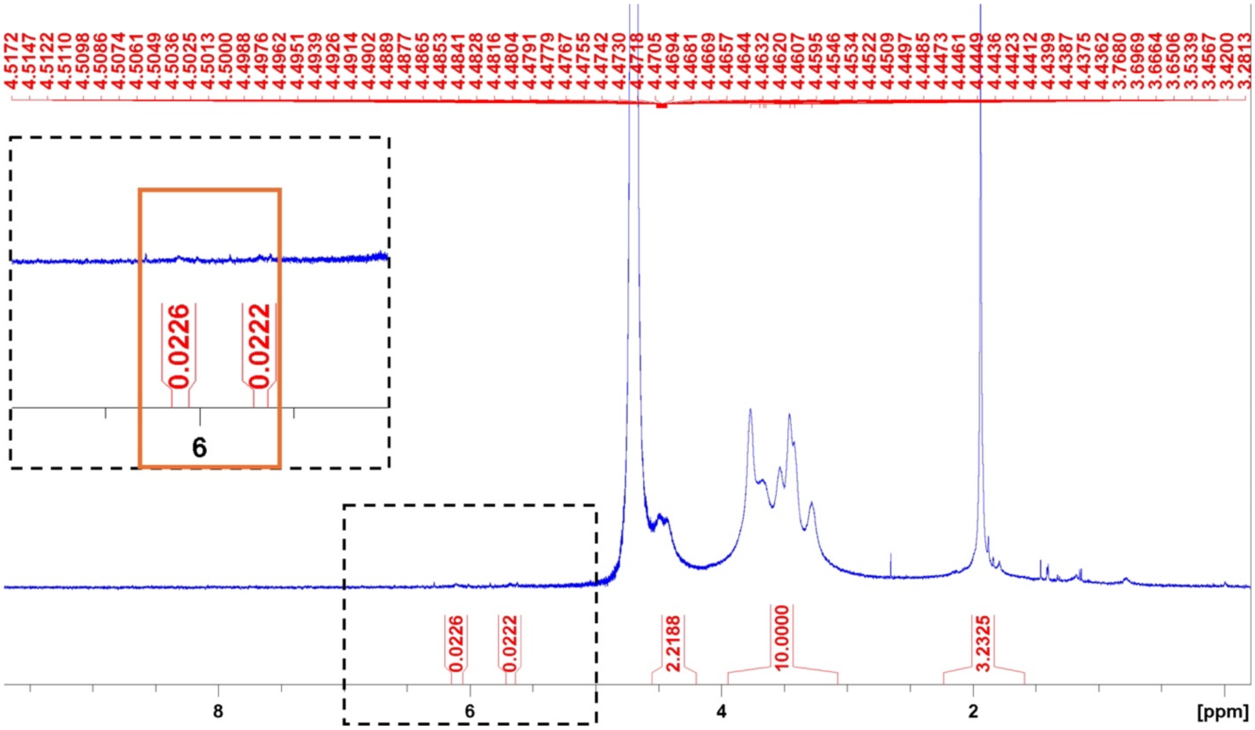

Figure S5. ^{1}HNMR Spectrum of HA-MA recorded in D_2O at 25 °C. The box shows an expansion of the region from 4.25-6.5ppm with the methacrylate protons of the HA-MA marked by the orange box.

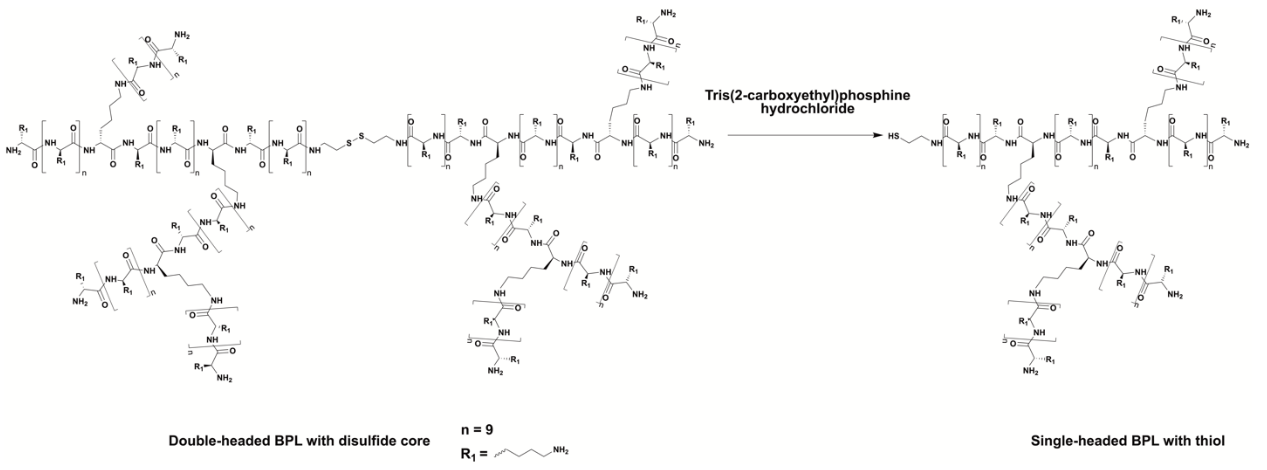

Figure S6. Reduction of disulfide bond in the double-headed BPL molecule.

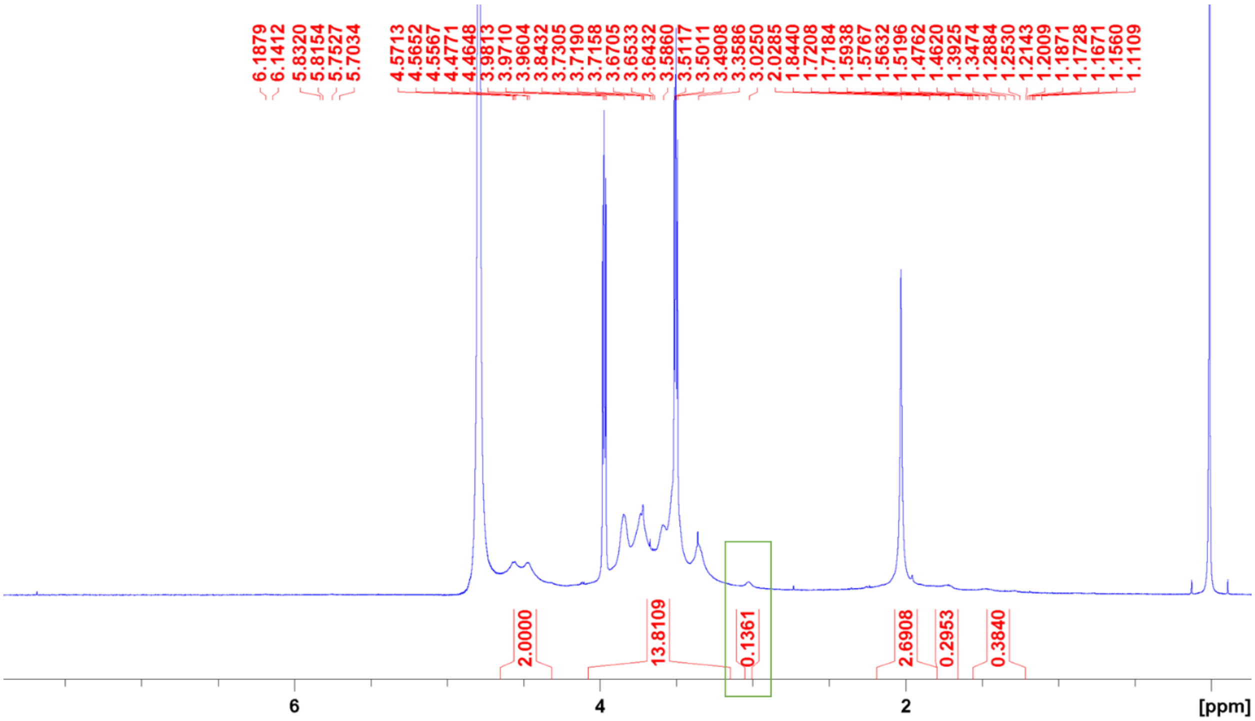

Figure S7. ^{1}HNMR Spectrum of HA-2BPL recorded in D_2O at 25 °C. Peak at 3.025 ppm (marked with a green colored box) indicates the appearance of methylene protons adjacent to NH_2 group ($-CH_2CH_2CH_2CH_2NH_2$) of lysine unit from fully deprotected BPL.

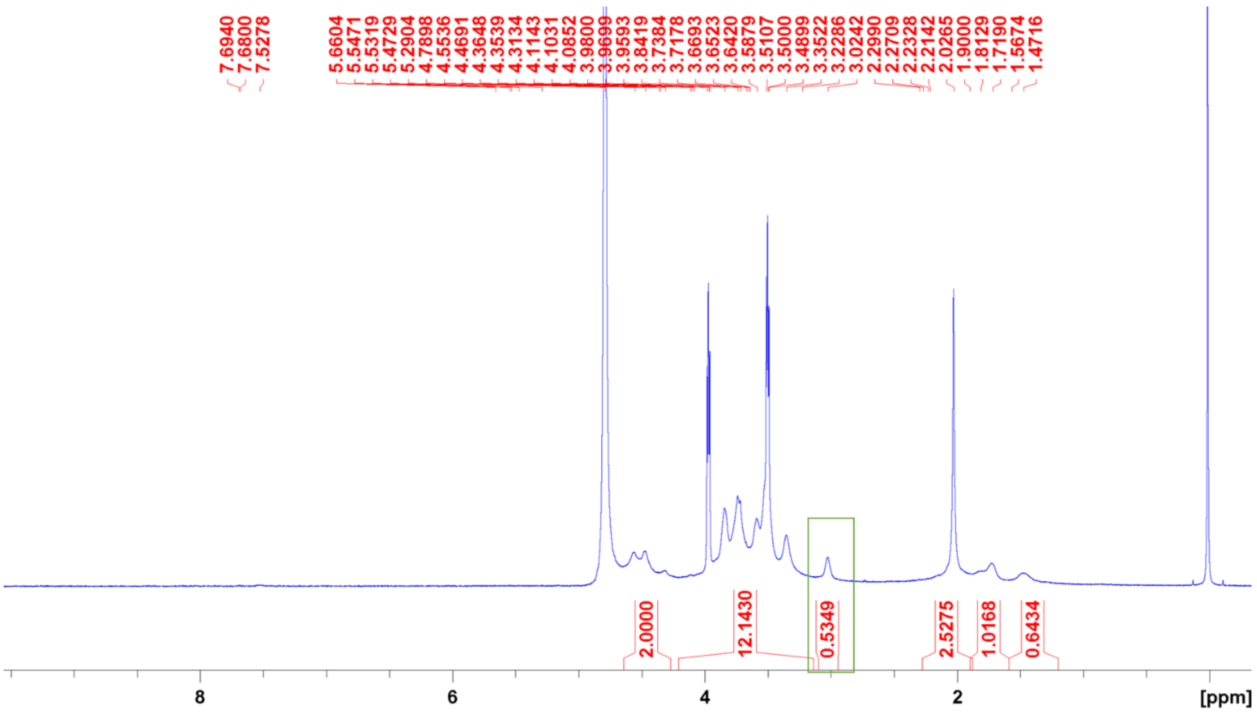

Figure S8. ^{1}HNMR Spectrum of HA-8BPL recorded in D_2O at 25 °C. Peak at 3.024 ppm (marked with a green colored box) indicates the appearance of methylene protons adjacent to NH_2 group ($-CH_2CH_2CH_2CH_2NH_2$) of lysine unit from fully deprotected BPL.

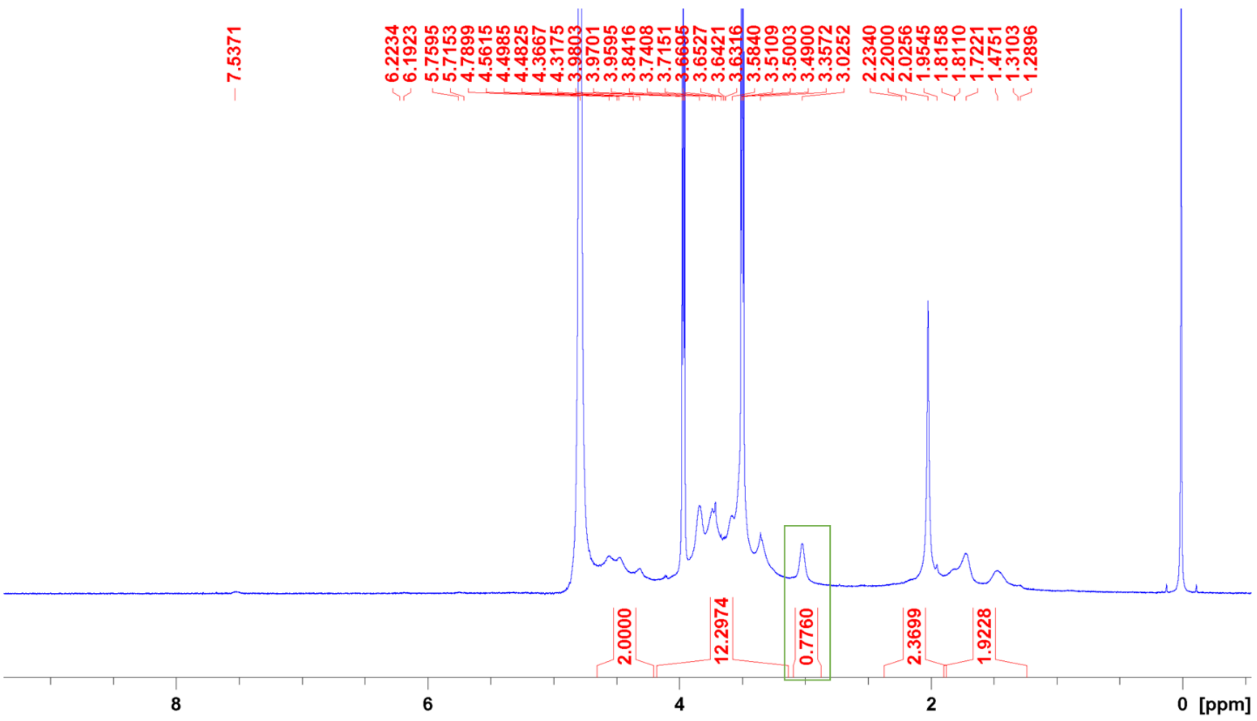

Figure S9. ^{1}HNMR Spectrum of HA-12BPL recorded in D_2O at 25 °C. Peak at 3.025 ppm (marked with a green colored box) indicates the appearance of methylene protons adjacent to NH_2 group ($-CH_2CH_2CH_2CH_2NH_2$) of lysine unit from fully deprotected BPL.

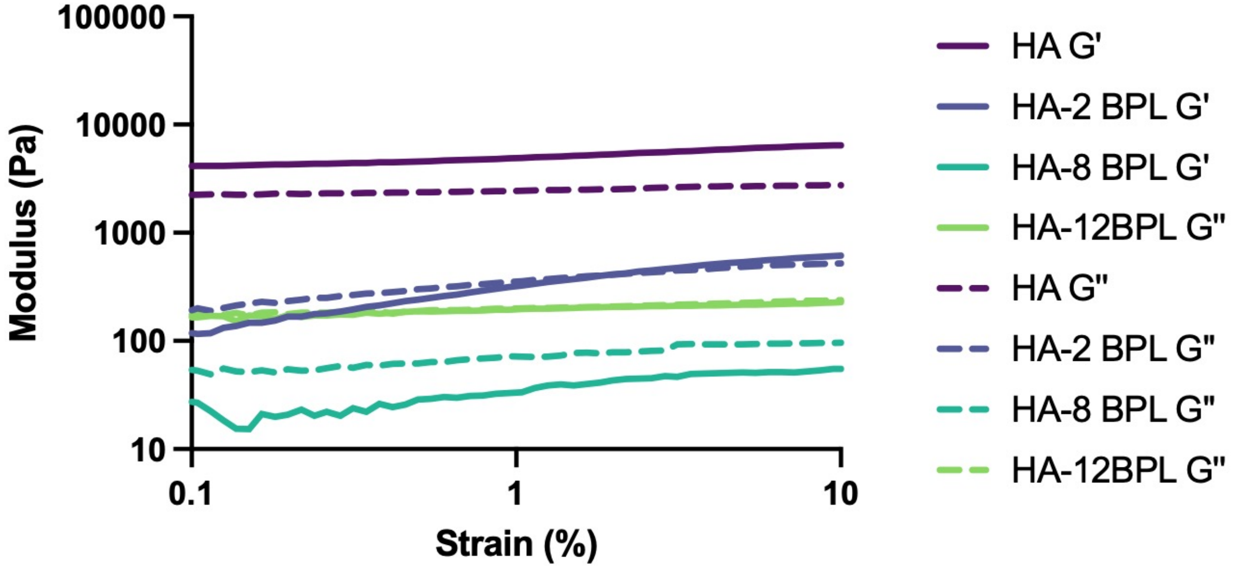

Figure S10. Strain sweep of HA and HA-BPL molecules.

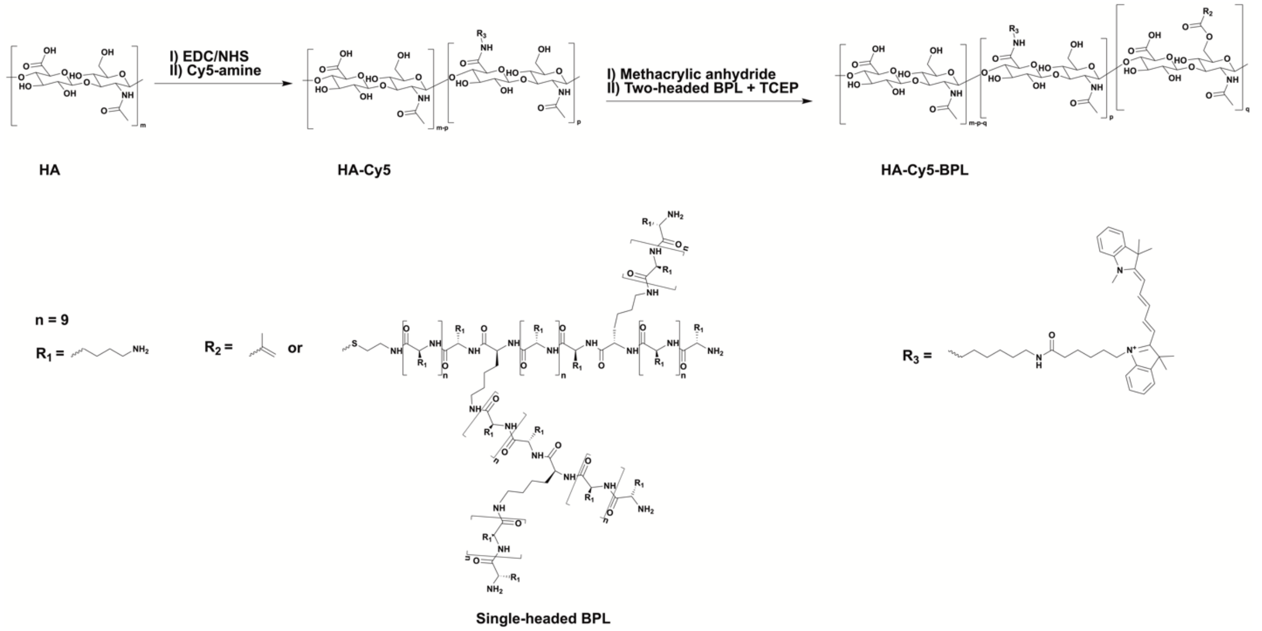

Figure S11. Synthesis of Cy5 containing HA-BPL molecules.

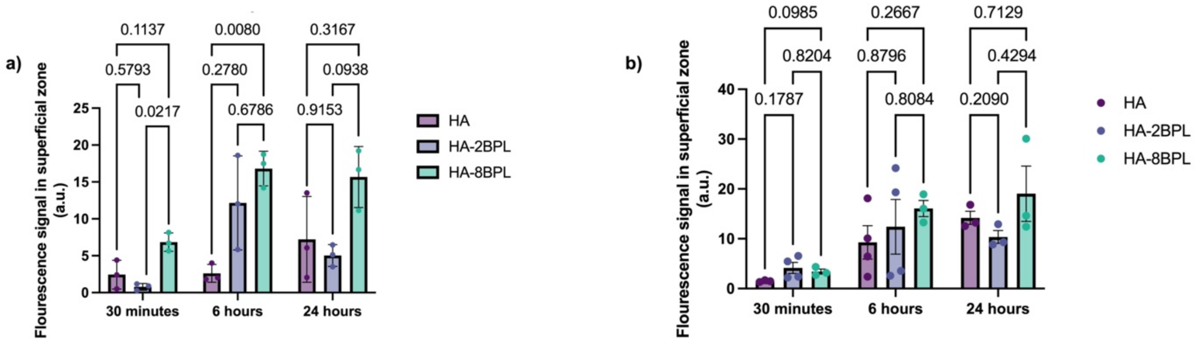

Figure S12. Adsorption of HA-BPL molecules to cartilage as a function of time. a) Penetration of HA BPL molecules in healthy cartilage explants. b) Penetration of HA BPL molecules in OA-mimetic cartilage explants.

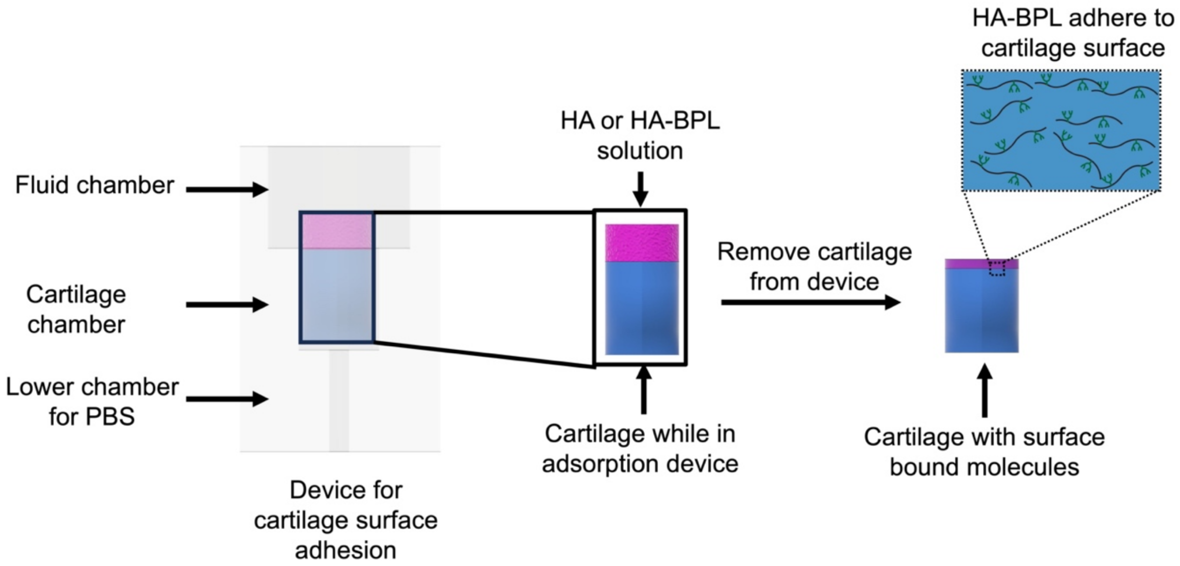

Figure S13. Schematic of device used to assess adsorption of HA and HA-BPL molecules to cartilage.

Table S1. Molecular characteristics of different generations

	Number of terminal lysine residues	Total number of branches/arms	[a]DP_{branch}	[b]DP_{total}
Core	2	2	10	20
G0	4	6	10	60
G1	8	14	10	140

[a,b] Theoretical estimation of average number of lysine repeating units per branch (DP_{branch}) and the total number average degree of polymerization (DP_{total})
[a] Yields determined gravimetrically after precipitation, filtration, and freeze-drying of the polypeptides.

Table S2. Results of the ^{1}HNMR characterization of BPL molecules

^{1}HNMR Integrals					
	$I(H_a) + I(H_b)$	$I(H_c)$	$[I(H_a)+ I(H_b)+ I(H_c)]/I(H_c)$	DP_{branch}	DP_{total}
Core	20.3	2	11.15	~11.1	22
G0	85.0	4	22.25	~11.1	89
G1	178.76	8	23.34	~11.6	187

$I(H_a)$ = Integral for methine protons of the terminal lysine units from the core peptide
$I(H_b)$ = Integral for methine protons of the terminal lysine units from the G0 peptide
$I(H_c)$ = Integral for methine protons of the terminal lysine units from the G1 peptide
